# Ultrafast consolidation of bulk nanocrystalline titanium alloy through ultrasonic vibration

**DOI:** 10.1038/s41598-018-19190-8

**Published:** 2018-01-15

**Authors:** P. Chen, W. B. Liao, L. H. Liu, F. Luo, X. Y. Wu, P. J. Li, C. Yang, M. Yan, Y. Liu, L. C. Zhang, Z. Y. Liu

**Affiliations:** 1 Guangdong Provincial Key Laboratory of Micro/Nano Optomechatronics Engineering, College of Mechatronics and Control Engineering, Shenzhen University, Shenzhen, 518060 China; 2 College of Physics and Energy, Shenzhen University, Shenzhen, 518060 China; 3 Department of Mechanical Engineering, Tsinghua University, Beijing, 100084 China; 4 National Engineering Research Center of Near-net-shape Forming for Metallic Materials, South China University of Technology, Guangzhou, 510640 China; 5 Department of Materials Science and Engineering, South University of Science and Technology, Shenzhen, 518055 China; 6 State Key Laboratory of Powder Metallurgy, Central South University, Changsha, 410083 Hunan China; 7 School of Engineering, Edith Cowan University, 270 Joondalup Drive, Joondalup, Perth, WA 6027 Australia

## Abstract

Nanocrystalline (NC) materials have fascinating physical and chemical properties, thereby they exhibit great prospects in academic and industrial fields. Highly efficient approaches for fabricating bulk NC materials have been pursued extensively over past decades. However, the instability of nanograin, which is sensitive to processing parameters (such as temperature and time), is always a challenging issue to be solved and remains to date. Herein, we report an ultrafast nanostructuring strategy, namely ultrasonic vibration consolidation (UVC). The strategy utilizes internal friction heat, generated from mutually rubbing between Ti-based metallic glass powders, to heat the glassy alloy rapidly through its supercooled liquid regime, and accelerated viscous flow bonds the powders together. Consequently, bulk NC-Ti alloy with grain size ranging from 10 to 70 nm and nearly full density is consolidated in 2 seconds. The novel consolidation approach proposed here offers a general and highly efficient pathway for manufacturing bulk nanomaterials.

## Introduction

Once the grain size is refined to nanometer scale, the polycrystalline materials tend to exhibit excellent physical and chemical properties^1–5^. Hence, the development of feasible approach to fabricate bulk nanocrystalline (NC) materials has been desired over decades^6,7^. One approach is to refine grains by severe plastic deformation (SPD), which involves a complex stress state resulting in equiaxed grains with high-density dislocations. However, the SPD method is applicable most for ductile and low-strength materials, such as aluminum, magnesium and copper alloys. And the minimum grain size is hard to be refined less than 100 nm due to the temperature rise during severe deformation^8–10^. Consolidation of NC powder is another route for synthesizing bulk nanomaterials^11–15^. The powder related approach often requires lengthy heating time or high sintering temperature, which is prone to result in severe grain coarsening of the sintered components^16,17^. Hitherto, it is still difficult to fabricate bulk fully dense NC materials in high-strength alloy systems. As one of material joining techniques, ultrasonic welding has high heating rate caused by extremely rapid friction at contacting interface of the workpieces. In consequence, it has being widely used to weld low-melting-point materials, including plastics, polymers and some non-ferrous metals^18–20^. Recently, ultrasonic vibration has also been employed as an assisted heating method to improve the densification of the compacts during consolidation^21–23^. At the meantime, as a kind of peculiar materials with metallic bond and glassy atomic packing structure^24^, metallic glasses (MGs) exhibit unique softening behavior above their glass transition temperature *T*_*g*_, at about 0.55 of melting temperature *T*_*m*_. This significantly reduces the processing threshold of MGs^25,26^. In addition, above *T*_*g*_ the viscous flow of the supercooled liquid can accelerate the consolidation process and retard the nucleation and crystallization of the MG powders (MGPs)^27–29^. Based on these properties, a new method, ultrasonic vibration consolidation (UVC), is developed to fabricate bulk NC-Ti alloy starting from the MGPs precursor. Bulk specimens with equiaxed two-phase nanostructure and high relative density are obtained after 2 seconds consolidation. The novel approach proposed here offers a general and highly efficient pathway for manufacturing bulk NC materials.

## Results and Discussion

**Figure 1:**
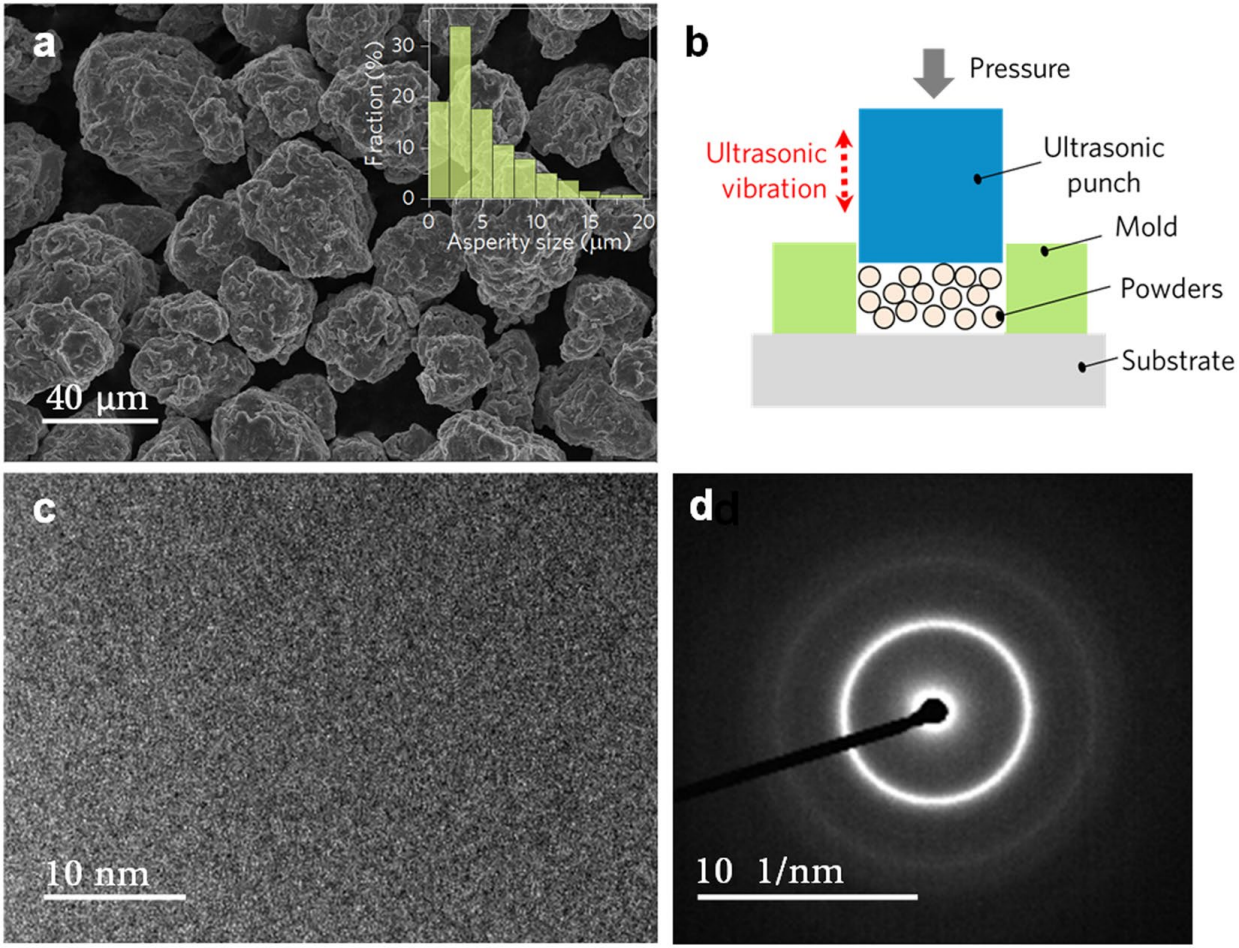
(**a**) Morphology of the Ti-based MGPs with many asperities formed during ball milling. (**b**) Schematic diagram of UVC method composed of punch vibrating at ultrasonic frequency, mold, substrate and powders. (**c**) TEM image of the ball-milled Ti-based MGP. (**d**) Selected-area electron diffraction (SAED) pattern of the powder.

**Figure 2:**
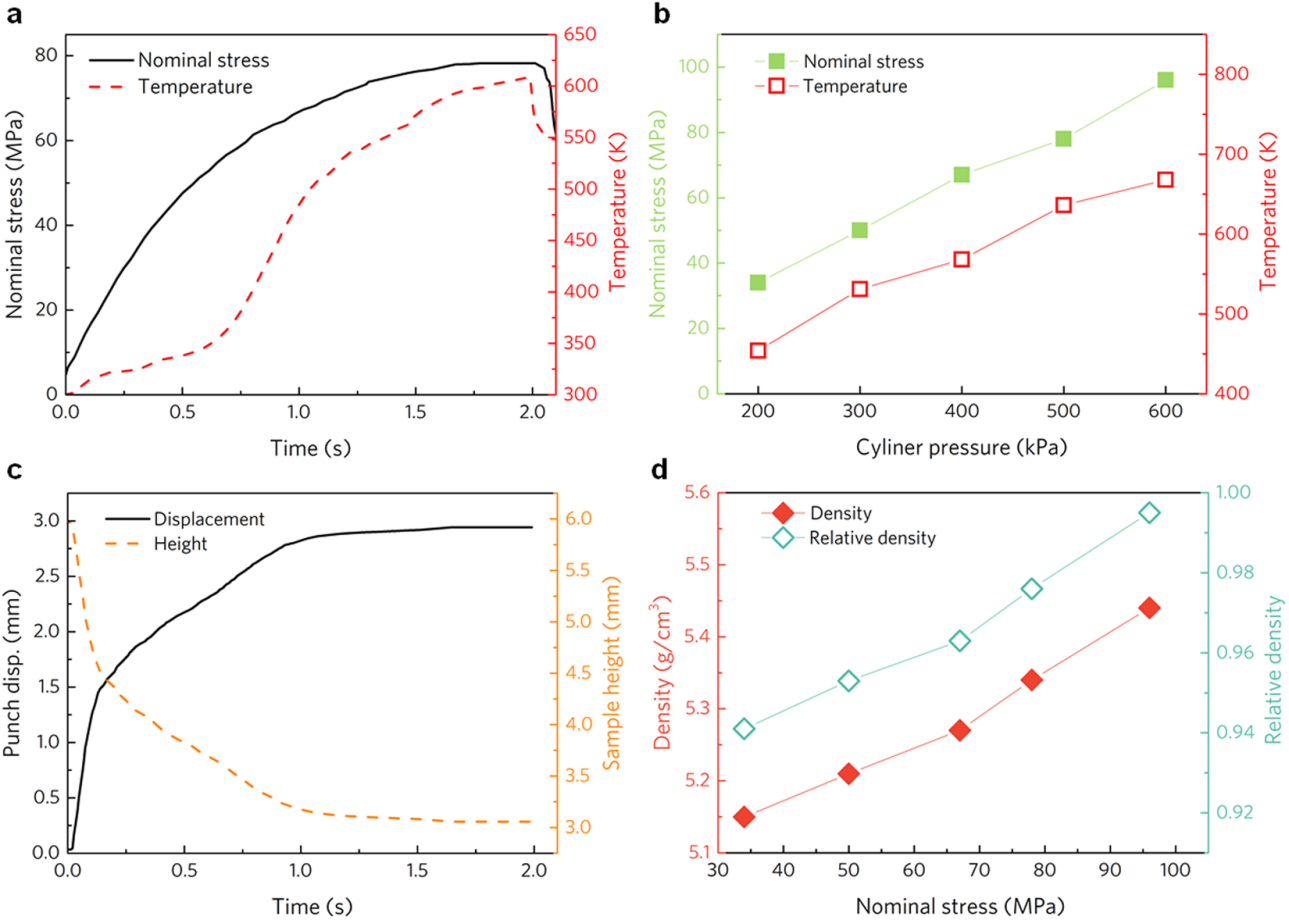
Ultrasonic consolidation process under different cylinder pressure. (**a**) Typical nominal stress-time-temperature curves under cylinder pressure of 500 kPa. (**b**) The maximum nominal stress and temperature under different cylinder pressure. (**c**) Typical punch displacement-time-sample height curves under cylinder pressure of 500 kPa. (**d**) Density and relative density of the consolidated bulk Ti alloy obtained by different nominal stresses.

Morphological features of the ball-milled Ti_66_Nb_13_Cu_8_Ni_6.8_Al_6.2_ (at.%) powders are shown in Fig. 1a. The average diameter of the powders is measured to be about 20 μm. Due to the repeated collision and fracture during ball milling, the surface of the powders has different asperities, ranging from about 0.6 to 18 μm (Fig. 1a inset). A large proportion (33%) of the asperities fall around 3 μm. As the schematic diagram of the UVC approach presented in Fig. 1b, under the combined effects of pressure and ultrasonic vibration imparted by punch, the powders can be consolidated into a bulk form. XRD pattern of the ball-milled powders indicates the amorphous nature of the powders (not shown here), which is further confirmed by TEM image and corresponding selected-area electron diffraction (SAED) pattern, as shown in Fig. 2c and d.

Fig. 2a shows the typical nominal stress-time-temperature curves under preset cylinder pressure of 500 kPa. The nominal stress *σ*_*n*_, obtained by dividing the applied force *F* by the cross-sectional area *A* of the compact, increases with time until a full load of 78 MPa at 1.78 s. The maximum loading rates under different preset pressure are listed in Table 1, where an increasing trend can be observed. For the measured temperature curve, in the initial period of time, the temperature rises slowly to a low platform at ~320 K. After a short dwell between 0.25 and 0.60 s, the temperature surges rapidly to a high platform, corresponding to the preset full load. The maximum heating rate is extracted from the curve and it increases from 1.66 × 10^4^ K/min at 200 kPa to 8.55 × 10^4^ K/min at 600 kPa, which is much higher than that of traditional fast sintering methods, such as 1 × 10^3^ K/min of spark plasma sintering^30^, 5 × 10^3^ K/min of microwave sintering^31^, implying the capability of UVC to avoid grain coarsening. The full nominal stress and maximum temperature for different preset cylinder pressures are shown in Fig. 2b. It can be found that both the nominal stress and the measured temperature increase linearly with the cylinder pressure, from 34 to 97 MPa for the nominal stress and from 454 to 668 K for the temperature, respectively. In addition, based on the data of punch displacement recorded during the compaction, the instantaneous sample height is obtained by subtracting punch displacement from the initial powder-filling height. As shown in Fig. 2c, the change in punch displacement follows the similar trend as that of the nominal stress during consolidation except there is a turning point at ~0.5 s for the curve slope, the discrepancy in force and displacement suggests certain transformation takes place in the consolidated powders. The instantaneous height of specimen shows the opposite trend, and final specimen height is about 3 mm. Density of the consolidated specimens were measured. It is found that the density increases almost linearly from 5.149 to 5.454 g/cm^3^ with the increase of nominal stress. The relative density is calculated from the ratio of the consolidated density to the theoretical density (5.470 g/cm^3^) of the Ti_66_Nb_13_Cu_8_Ni_6.8_Al_6.2_ alloy^32^, and it is found that a high relative density of 99.7% is achieved by employing the novel UVC method within 2 seconds under preset nominal stress 97 MPa. The consolidation time of the UVC is two orders of magnitude smaller than that of fast sintering methods^30,31^, demonstrating its high efficiency for the powders densification.

**Table 1:** Consolidation parameters of the UVC process and structural information of the consolidated bulk specimens.

Cylinder Pressure (kPa)	Maximum loading rate (N/s)	Maximum heating rate (10^4^ K/min)	Measured temperature (K)	Lattice constant of β Ti (Å)	Density (g/cm^3^)	Relative density (%)
200	999	1.66	454	3.263	5.149	94.1
300	1337	1.85	531	3.263	5.213	95.3
400	1683	1.94	568	3.266	5.267	96.3
500	1838	2.62	637	3.272	5.337	97.6
600	2265	8.55	668	3.268	5.454	99.7

**Figure 3:**
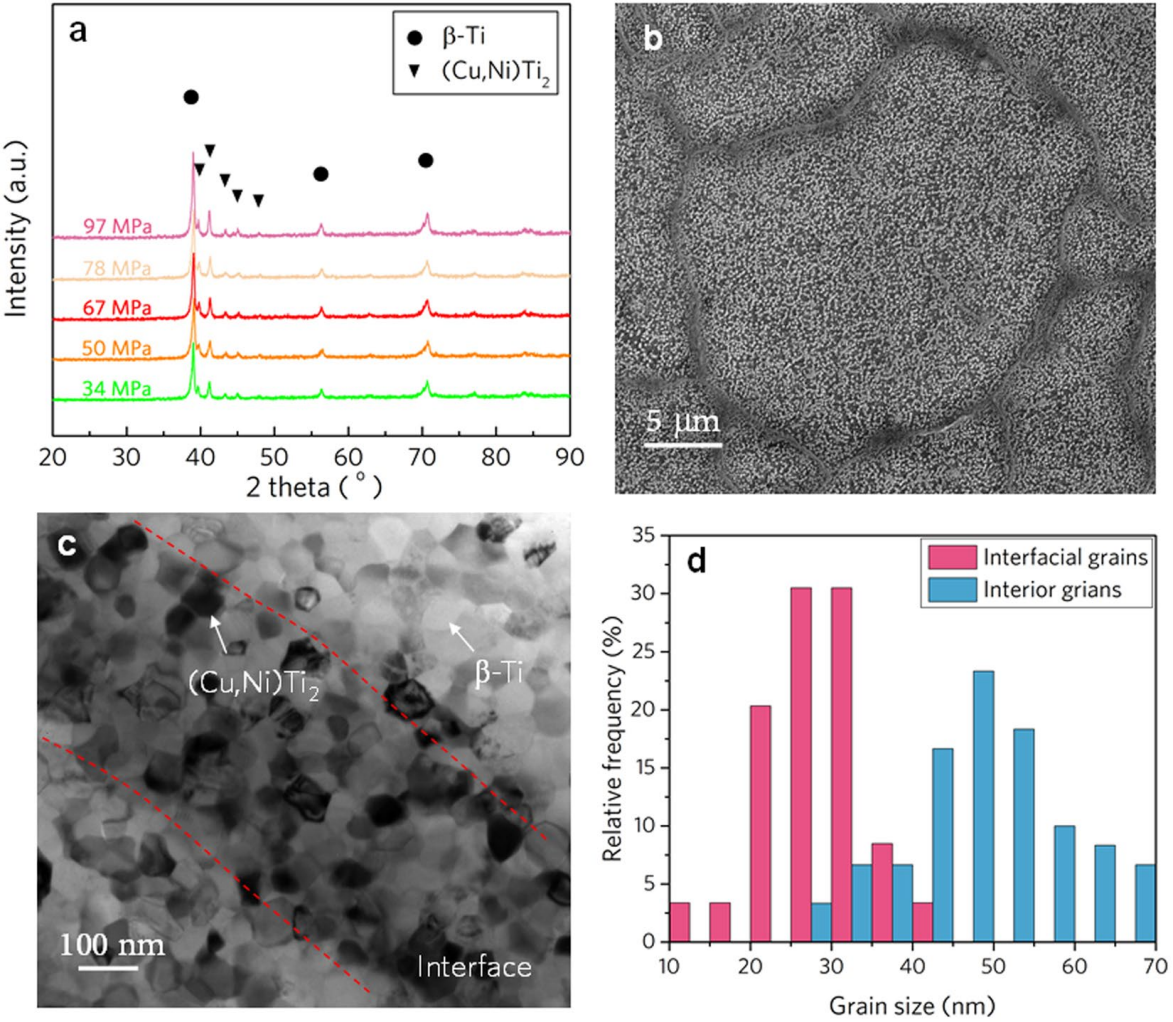
Microstructures of the UVC consolidated NC-Ti alloy. (**a**) XRD patterns of the consolidated NC-Ti alloy. (**b**) SEM image of the NC-Ti alloy with distinct boundary between the original powders. (**c**) TEM image of the interface and interior regions of the NC-Ti alloy. (**d**) The grain size distributions in the interface and interior region of Fig. 3c.

Fig.3a shows the XRD patterns of the consolidated specimens. It suggests that significant crystallization occurs in the consolidation process. The crystallized bulk specimens are mainly constituted by β-Ti phase with a body-centered cubic (bcc) structure and a secondary (Cu,Ni)Ti_2_ phase with a face-centered cubic (fcc) structure, which are similar with previous studies^32,33^. It is also found that the lattice constant of the β-Ti increases from about 3.263 to 3.268 Å with the increase in pressure, slightly higher than those obtained by spark plasma sintering^33^. This implies shorter diffusion time for the formation of β-Ti during UVC under higher heating rate. Fig. 3b presents the microstructure of the near fully dense specimen consolidated under 600 kPa, it is observed that some nanoscale particles are precipitated in the matrix. And it also can be found that well-defined interface layer with thickness of hundreds nanometers is located between the precursor powders, suggesting that some kind of fusion occurs at the adjoin region during consolidation. High resolution TEM image of the interface region is shown in Fig. 3c, illustrating that the consolidated Ti alloy has a clean equaxied nanocrystalline structure with grains size ranging from 10 to 70 nm with an average value at about 39 nm. This is one of the smallest grain sizes of titanium alloys among the previously-reported data obtained by sintering or SPD^33–35^. A closer examination reveals that the interface region has a relative darker contrast and thickness of about 400 nm. Statistical analyses of the grain size distributions in the interface and interior regions unveil that the interface is composed of smaller grains ranging from 13 to 42 nm with an average grain size of about 29 nm, while the interior region of the powder has much larger grains ranging from 28 to 69 nm with an average size of about 49 nm, which is 70% larger compared with the interface region, as shown in Fig. 3d. The distinctly different grain size distribution indicates that the interface region between the powders experiences different thermal processes during consolidation.

**Figure 4:**
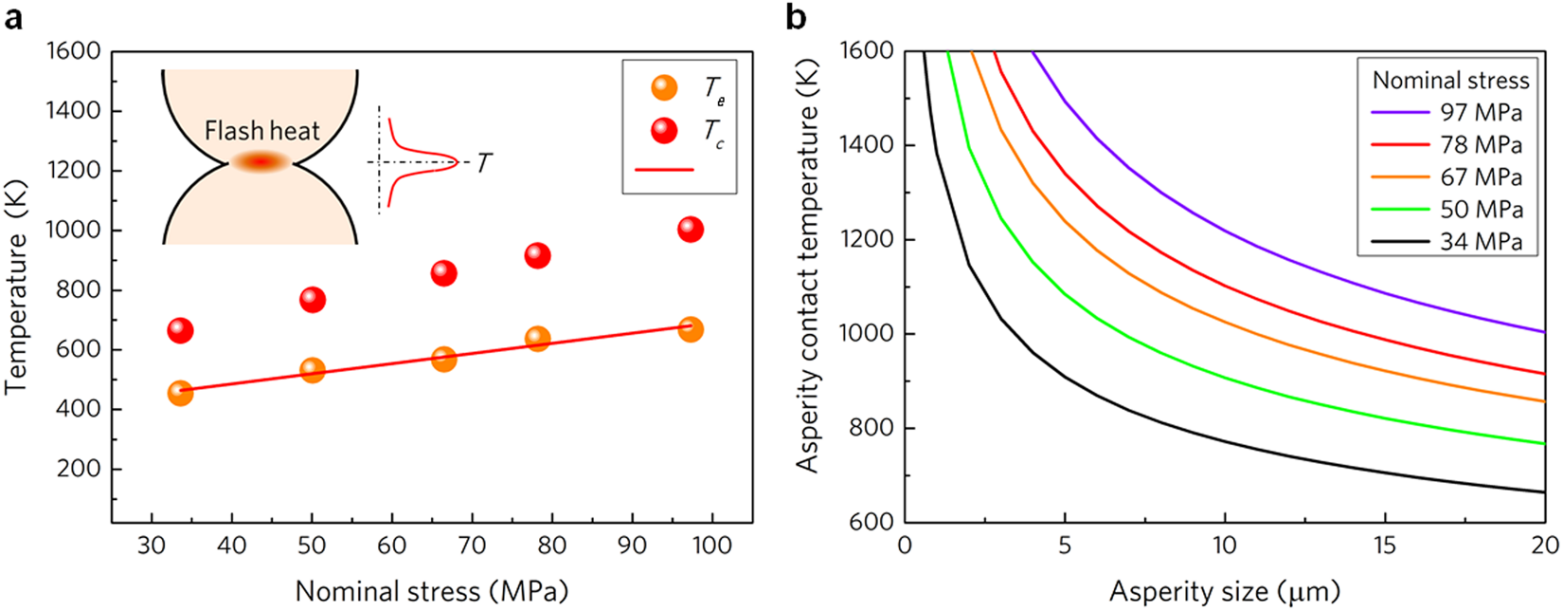
Frictional heating mechanism during the UVC. (**a**) Temperature measured by thermocouple and theoretical contact temperature generated by frictional flash heat. (**b**) Asperity contact temperature as a function of the asperity size on the surface of powder.

UVC bonds the adjacent powders together under the combined action of force and mechanical vibration, thereby making the approach to some extent analogous to the ultrasonic welding. It is reported that the heat of ultrasonic welding originates from friction between workpieces and anelastic deformation energy dissipation of the amorphous structure^19^. For UVC, the experimental measured maximum temperature using thermocouple *T*_*e*_ is proportional to the applied nominal stress, as shown in Fig. 4a. Linear fitting gives: *T*_*e*_ = *T*_*i*_ + *cσ*_*n*_, where *T*_*i*_ is the initial step temperature (extracted to be about 350 K), corresponding to the low temperature platform in Fig. 2a, *c* is a constant involving the friction coefficient and thermal parameters of the alloy (extracted to be 3.4 K/MPa). As the anelastic energy is proportional to the square of the stress, such a linear relationship indicates that friction dominates the heating process of UVC.

However, the measured temperature is much lower than *T*_*g*_ (*T*_*g*_ = 715 K) of the MGP^36^, then how the powders can be bonded together in solid state during UVC process? In order to further reveal the underlying mechanism, the single joint region of the powder compact is concerned (Fig. 4a inset). Assuming that the powder has an ideal spherical shape, the local stress applied at the contact point would be much higher than the nominal stress due to stress concentration. The contact stress *σ*_*c*_ thus can be calculated using the equation:

1 $${\sigma }_{c}=\frac{3{F}_{i}}{2\pi {a}^{2}}$$

where *F*_*i*_ is the force taken by individual powder, *a* is the contact radius, can be given by the expression:

2 $$a=\sqrt[3]{\frac{3{F}_{i}}{16}\frac{(1-{\nu }^{2})d}{E}}$$

where *v, E* and *d* are the Poisson’s ratio (0.33), elastic modulus (110 GPa) and average diameter (20 μm) of the MGPs, respectively. It can be calculated that the contact radius ranges from about 600 to 900 nm at different preset pressure, resulting in the maximum contact stress larger than 10 GPa. Besides, the high speed vibration induces rapid rubbing between powders, the friction converts kinetic energy into thermal energy, leading to flash heating at the contact area. The heat remains only in the instant when the two neighboring powders are being in rubbing contact, thus, it can be called as flash heat^37^. And it is widely accepted that the flash temperature rise could be very high but only last less than 10^−3^ s, which makes the embedded thermocouple hard to capture the exact flash temperature owing to its limited thermal capacity and the distance from the points of intimate contact where heat is being generated. According to the friction theory^38^, the maximum flash temperature rise can be approximated by using the equation:

3 $${\rm{\Delta }}{T}_{f}\approx \frac{2qa}{\kappa \sqrt{\pi (1.273+{P}_{e})}}$$

where *q* is the rate of heat generated per unit area of the contact between powders, given by *q* = *μvσ*_*c*_, *μ* is the coefficient of friction, taken as 0.25, *v* = *A*_*vib*_*/t*_*vib*_ is the relative friction speed calculated to be 3 m/s, *A*_*vib*_ and *t*_*vib*_ is the amplitude and time of the single vibration route, *κ* is the thermal conductivity (6.7 W/m∙K), *P*_*e*_ = *va*/2*k*, is the Péclet number, a dimensionless parameter relevant in the study of transient phenomena, *k* is the thermal diffusivity (2.3 × 10^−6^ m^2^/s). As seen in Fig. 4a, theoretically calculated contact temperature (*T*_*c*_ = *T*_0_ + *ΔT*_*f*_) during consolidation increases from 657 K to 1004 K with the increase in applied nominal stress, which is about 200~400 K higher than the corresponding experimental measured temperature *T*_*e*_. To be specific, at a stress of 34 MPa the calculated contact temperature *T*_*c*_ is 664 K, approaching to *T*_*g*_. While at stress of 50 MPa, *T*_*c*_ reaches 767 K, exceeding the *T*_*g*_ but lower than *T*_*x*_ *(T*_*x*_ = 799 K) of the Ti-based MGPs.

Dig a step further, let’s consider the specific morphological details of the powders as shown in Fig. 1a, the roughness on the powder surface could create much smaller contact area than the ideal smooth spherical powder. Therefore, lager contact stress, and consequent higher frictional heat can be naturally generated. Using the above equations, the contact temperature caused by asperities is estimated. As presented in the Fig. 4b, the asperity below 8 μm can induce a contact temperature higher than *T*_*x*_ at full stress of all the preset pressures. Most asperity falls into this size range (about 80% as shown in Fig. 1a inset). The analysis directly clarifies why the consolidation and crystallization take place during UVC process.

It is worth mentioning that the maximum contact temperature caused by asperity is calculated based on the assumption that the friction coefficient remains constant regardless of the temperature rise. This leads to the contact temperature rising to infinity with the further decrease in the asperity size, as shown in Fig. 4b. However, when the MG transforms to supercooled liquid state above *T*_*g*_, unlubrication friction becomes invalid, and further increase in temperature is retarded^39^. Furthermore, based on the contact temperature caused by flash heating, the maximum heating rate can be estimated to be as high as 4 × 10^7^ K/min, which is 4 orders of magnitude higher than that of traditional fast sintering^30^. Such a high heating rate facilitates the fabrication of nanocrystalline materials, as demonstrated in Fig. 3c. The nanocrystallization of MG involves crystalline nucleation and growth, the final size of the grain reflects the time and temperature dependence of the both process. Because the heat is generated by the interfacial friction, the contact surface has the highest temperature peak (Fig. 4a inset). In deeply undercooled region, the higher interfacial temperature engenders much greater increase in nucleation rate than that in growth rate^40^, therefore resulting in finer grains in the interface region compared with that in the interior. The interfacial structure feature shown in Fig. 3c further confirms the above theoretical analysis.

**Figure 5:**
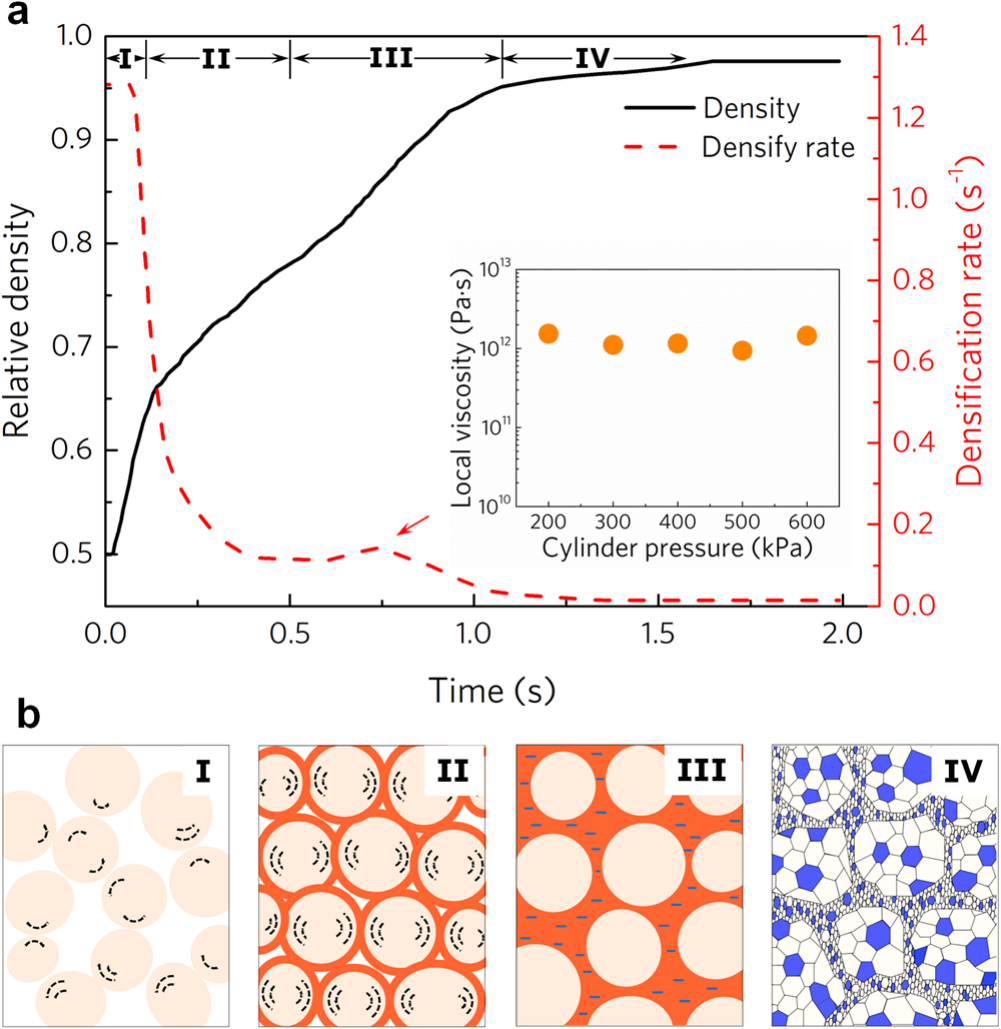
Consolidation mechanism of UVC process. (**a**) Densification process shows that consolidation can be divided into four stages, inset is the local viscosity during stage III. (**b**) Schematic diagram of the different stages during consolidation, including: I, rearrangement of powders. II, frictional heating. III, viscous flow induced by the flash heat. and IV, crystallization of the specimen.

Fig. 5a shows typical relative density-time-densification rate curves of the UVC process. Intuitively, the densification process can be divided into the following four stages. In stage I - initial rapid densification, the relative density increases rapidly from ~0.52 to 0.65, meanwhile, densification rate reduces dramatically from 1.3 to 0.7 s^−1^. In stage II - slowdown of the densification, densification rate further decreases from 0.6 to 0.11 s^−1^ and the relative density increases smoothly from 0.66 to 0.74. In stage III - accelerated densification stage, the densification rate bounces back to 0.14 s^−1^, with relative density increasing from 0.74 to ~0.90. During this stage, the peak strain rate can be extracted to be between 0.4 and 0.9 s^−1^ under different contact stress, the local viscosity then can be calculated as:

4 $${\eta }_{l}=\frac{{{\rm{\sigma }}}_{l}}{{\dot{{\rm{\varepsilon }}}}_{l}}\approx \frac{{{\rm{\sigma }}}_{c}}{{c}_{d}{c}_{i}\dot{\varepsilon }}$$

where *η*_*l*_, *σ*_*l*_, ${\dot{{\rm{\varepsilon }}}}_{l}$ is the local viscosity, stress and strain rate at the interface region between the powders, respectively; *c*_*d*_ is the density coefficient in consideration of free spaces in the specimen, taken as relative density value; *c*_*i*_ is the local interface coefficient, as deformation is mainly concentrated at the contact interface, *c*_*i*_ *c*an be taken as *c*_*i*_ = *δ/d*, where *δ* is thickness of the interface (shown in Fig. 3b). The calculated local viscosities under different preset cylinder pressures all fall around 10^12^ Pa·s (as presented in Fig. 5a inset), it clearly indicates that glass transition occurs at the interface of the MGPs during the accelerated densification stage of UVC. Afterward, in stage IV - final stage, the densification rate decreases to near zero and relative density increases to the final value at the end.

Based on the above analyses, a schematic diagram of the consolidation process involving powder rearrangement, frictional heating, viscous flow and final crystallization during UVC can be drawn, as shown in Fig. 5b. In stage I, the MGPs rearrange from simple random-arrangement structure to random-densely-packed structure under the mechanical disturbance exerted by the ultrasonic punch. Caused by plenty of vacant spaces between the powders, the initial densification rate is very high as shown in Fig. 5a. Along with the decrease in the porosity, the densification rate reduces dramatically. In stage II, random-densely-packed powders gradually transform to full dense-packed state. The contact number of each powder increases to maximum value^41^, resulting in relatively more effective frictional heating and all the surfaces of the powders begin to be heated up. This in turn could increase the densification rate of the specimen, and counteract the effect of the free space consumption of the compact. Therefore, the densification rate are no longer decreasing at the end of this stage. Due to the flash heating, in stage III, the contact temperature rises to *T*_*g*_ of the Ti-based MGPs, and the surfaces of the MGPs transform to supercooled liquid state, as shown in Fig. 5b III. Consequently, accelerated viscous flow dominates this stage and there is a densification rate bounce back. Finally in stage IV, additional flash heat gives rise to further heating and leads to the crystallization of the MGPs. As a result of the increase in viscosity after crystallization, consolidation kinetics slows down^42,43^. At the end, due to the conjunction of the powders, flash friction loses its physical origin, the specimen begins to cool down. NC structure with finer interface grains and relative larger interior grains is obtained, as shown in Fig. 5b IV.

The above discussion demonstrates that interfacial friction has a dominant role to heat the powders to supercooled liquid state during the ultrafast consolidation. Afterwards, any additional heat would readily lead to further crystallization of the metastable supercooled liquid^44^. Due to the flash heating of friction the consolidation is achieved in a remarkably short period of time, there is very limited time for the newly formed crystalline grains to grow. As a result, bulk nanocrystalline materials can be fabricated. Besides, it is also reported that ultrasound can also refine the grain during solidification by cavitations-induced heterogeneous nucleation^45,46^, similar mechanism might takes effect during the UVC, which need further investigation.

It is widely accepted that crystalline metallic powders are bonded together mainly by atom diffusion (e.g. volume diffusion and grain boundary diffusion) during sintering. For MGPs, the sintering mechanism is dominated by viscous flow like other glassy materials^27,47^. When the MG is heated to the supercooled liquid state, the densification rate could be greatly accelerated due to the drastic reduction of the viscosity *η*, which is at magnitude ~10^12^ Pa∙s at *T*_*g*_. The ultrafast heating induced by UVC coupled with accelerated viscous flow of the MGPs jointly create a novel high-efficiency route for consolidation.

Compared with main approaches for the fabrication of bulk NC materials, the SPD introduces a large amount of dislocations to refine the structure through plastic deformation. Thus SPD is applicable primarily to ductile metals. Traditional sintering is the process of consolidating powder to a bulk form by external heating, and the efficiency of external heat is low with heating rate ranging from 1 to 100 K/min. As such, it will take hours to complete the traditional sintering process. Even though spark plasma sintering has an internal heating (hating rate reaches up to 1000 K/min), the sintering time can be reduced to minutes, which is also too long to obtaining dense NC structure in many cases. The newly developed UVC takes advantages of the ultra-high frequency vibration and the ultrafast viscous flow of metallic glass at relatively lower temperatures to efficiently consolidate the MGPs and synthesize bulk nanocrystalline alloy. Furthermore, the ultrasonic vibration has already been widely utilized in industrial fields for the welding of various plastics. Based on our new findings that the UVC utilizes the ultrasonic friction as primary heating source, the method can be extended to sinter other glassy powders at low temperatures, such as polymer, inorganic glasses, and so on. Therefore, this work sheds light on the preparation of advanced bulk NC materials, and paves the way to exploit their higher performance.

In summary, an ultrafast consolidation method, namely ultrasonic vibration consolidation (UVC), is developed to fabricate bulk nanocrystalline titanium alloy using metallic glass powders as precursor. The heating mechanism is clarified as the frictional flash heat, which brings about ultrahigh heating rate at about 4 × 10^7^ K/min. The flash heating of UVC coupled with the accelerated viscous flow of the metallic glass lead to the rapid densification to approach the theoretical density in 2 s, therefore the UVC offers an efficient pathway to fabricate bulk nanocrystalline materials.

## Methods

MGPs with the nominal composition of Ti_66_Nb_13_Cu_8_Ni_6.8_Al_6.2_ (at.%) were synthesized from the mixture of elemental powders by mechanical alloying for 70 hours in a high-energy planetary ball mill (QM-2SP20, apparatus factory of Nanjing University) under a purified argon gas atmosphere. To consolidate the powders, a Branson ultrasonic welder 2000Xc with vibration frequency at 20 kHz and output amplitude at 75 μm was utilized. The welder possesses micrometer displacement resolution, millisecond time resolution and high output power 2500 W. The working part of the welder is mainly made up of air cylinder, piezoelectric converter, booster and punch. Among them, the air cylinder is the loading part, the piezoelectric converter converts high frequency electrical energy into high frequency mechanical motion, its amplitude is magnified by the booster, then the punch further amplifies the amplitude and transfers the pressure and vibration to the powders. The welder’s stack contracts and expands, resulting in longitudinal wave vibration at the punch with a simple harmonic waveform. The milled powders were filled into a mold with an inner diameter of 5 mm and a height of 6 mm. Five cylinder pressures ranging from 200 to 600 kPa were used to compact the powders, weld time with 2 s was selected to ensure fully loaded of the air cylinder, and ultrasonic vibration trigger force were set at 50 N for all the consolidations. At the same time, the temperature during the consolidation processes were measured using a thermocouple embedded in the bottom of the powders. Amorphous nature of the MGPs and the structures of the consolidated bulk specimens were characterized using a Rigaku SmartLab X-ray diffractometer with a Cu K*α* radiation source. Density of the consolidated specimens were measured according to Archimedes’ principle. The morphologies of the MGPs and the microstructure of the NC-Ti alloy were observed using a ZEISS SUPRA 55 Field Emission Scanning Electron Microscope (FE-SEM). The nanostructures were characterized using Transmission Electron Microscope (TEM) JEM–3200FS equipped with 300 kV field emission gun.
